# Supplementary figures and images for: Establishment of developmental gene silencing by ordered polycomb complex recruitment in early zebrafish embryos
Source: eLife. 2022 Jan 4;11:e67738. doi: 10.7554/eLife.67738 (PMC8769650; doi:10.7554/eLife.67738)

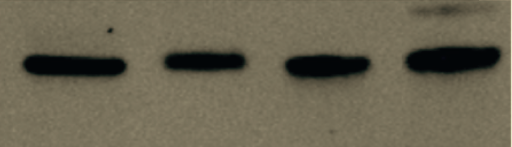

Supplement: Figure 1—source data 1. [file elife-67738-fig1-data1.zip › Figure_1_source_data/Figure_1_H3_blot.png]

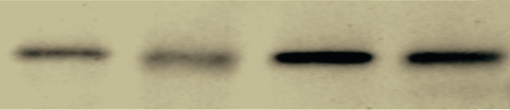

Supplement: Figure 1—source data 1. [file elife-67738-fig1-data1.zip › Figure_1_source_data/Figure_1_H2Aub1_blot.png]

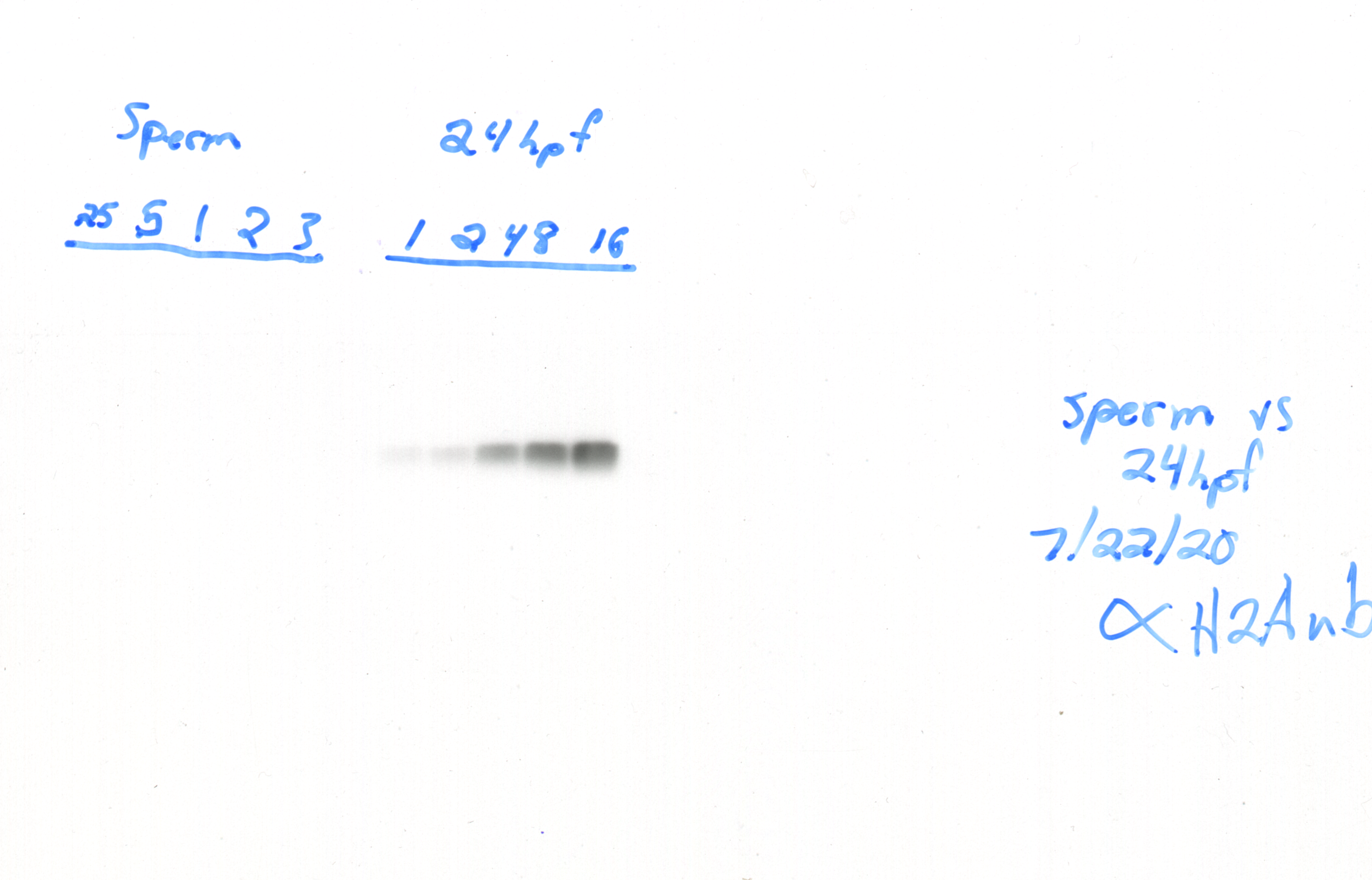

Supplement: Figure 1—figure supplement 1—source data 1. [file elife-67738-fig1-figsupp1-data1.zip › Figure_1_figure_supplement_1_source_data/H2Aub1_blot_short_exposure_Sperm_vs_24hpf.tif]

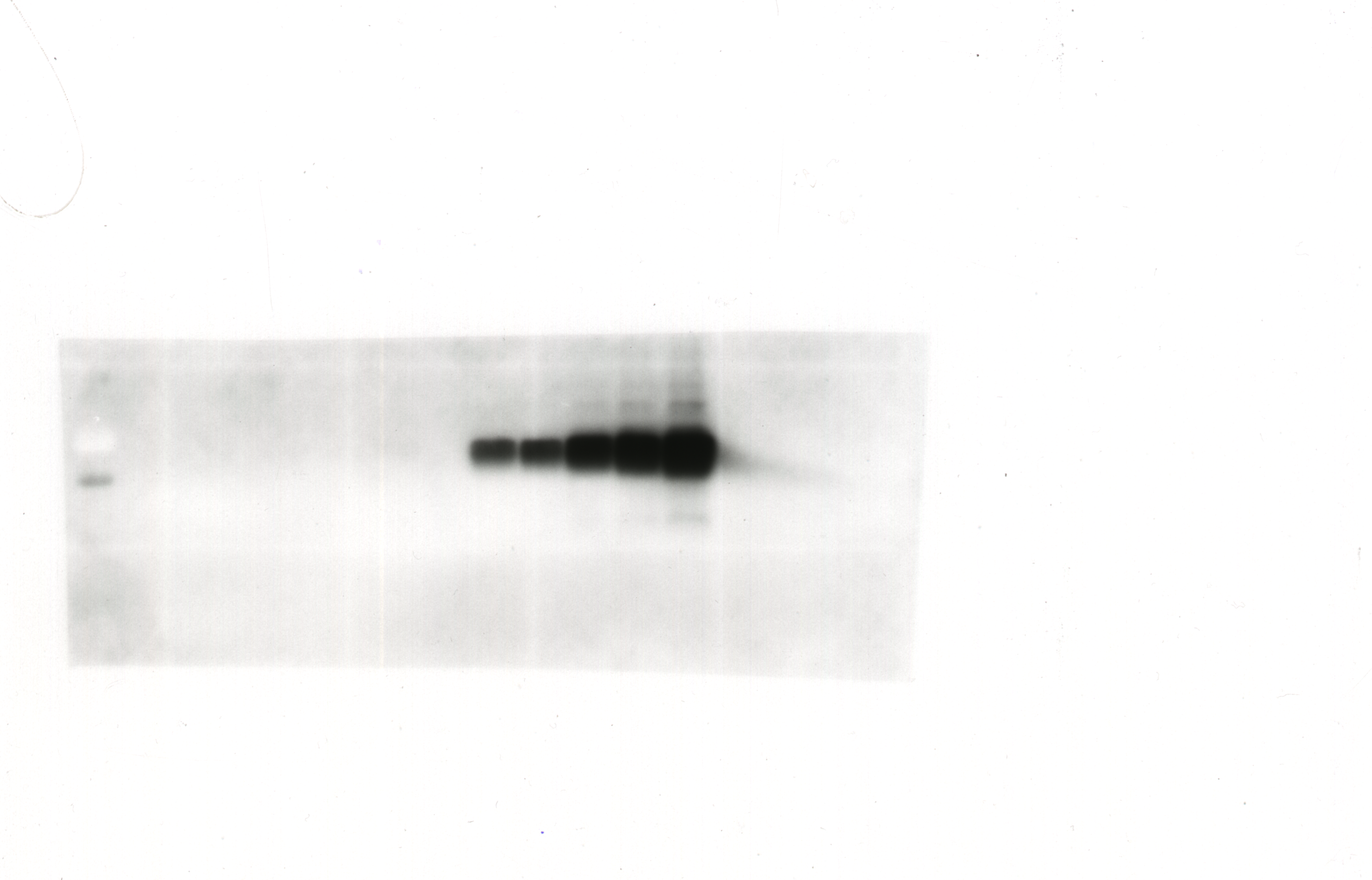

Supplement: Figure 1—figure supplement 1—source data 1. [file elife-67738-fig1-figsupp1-data1.zip › Figure_1_figure_supplement_1_source_data/H2Aub1_blot_long_exposure_Sperm_vs_24hpf.tif]

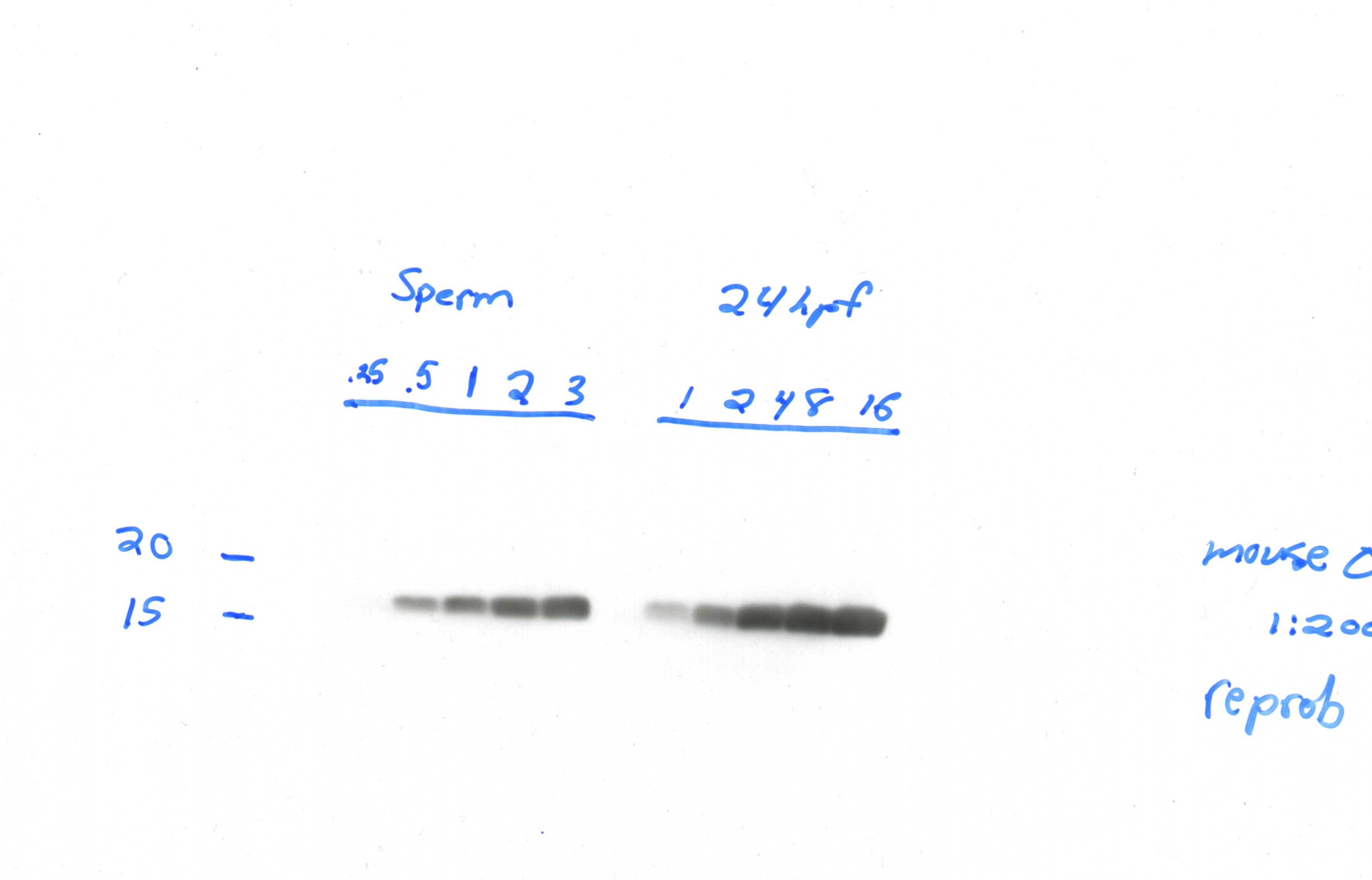

Supplement: Figure 1—figure supplement 1—source data 1. [file elife-67738-fig1-figsupp1-data1.zip › Figure_1_figure_supplement_1_source_data/H3_blot_Sperm_vs_24hpf.tif]

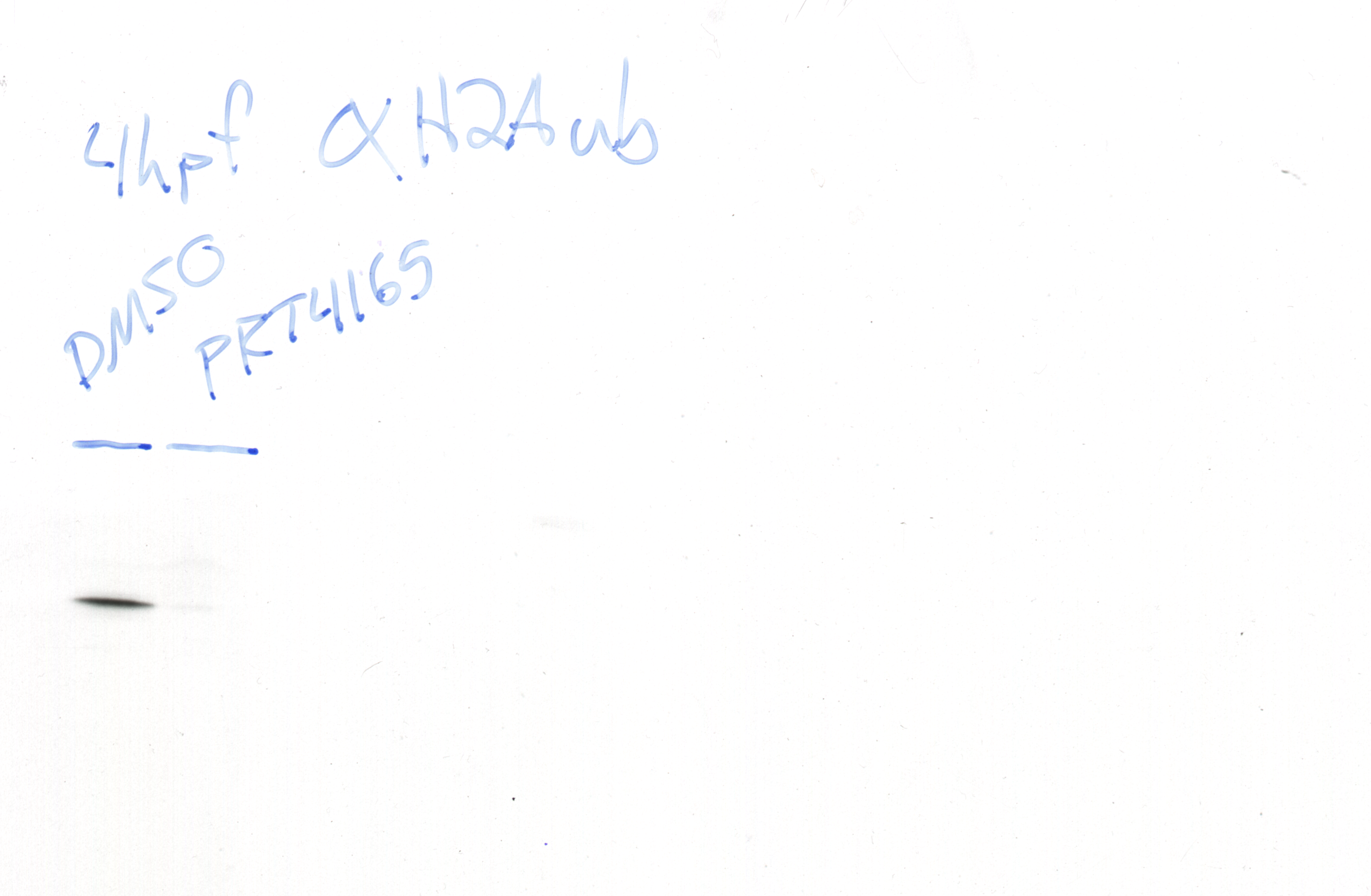

Supplement: Figure 4—source data 1. [file elife-67738-fig4-data1.zip › Figure4_source_data/H2Aub1_blot_DMSO_vs_PRT4165.tif]

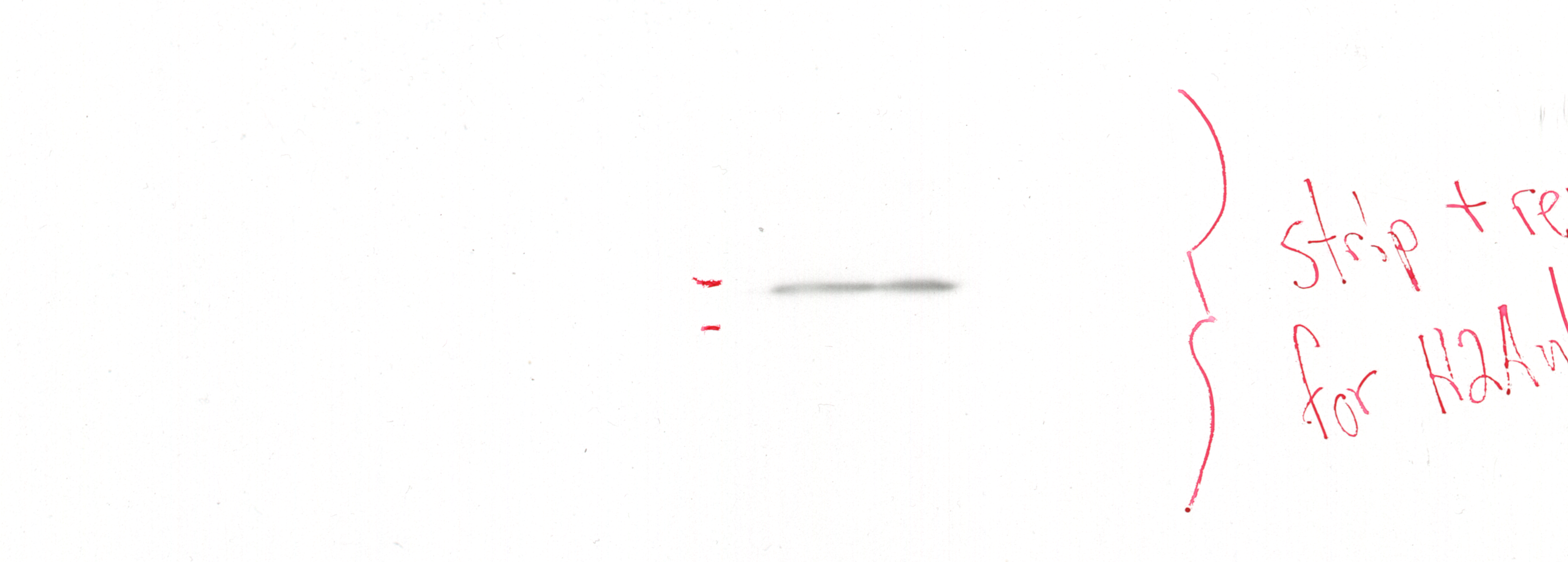

Supplement: Figure 4—source data 1. [file elife-67738-fig4-data1.zip › Figure4_source_data/H3_blot_DMSO_vs_PRT4165.tif]
